# Hematopoietic and Lymphoid Cell Neoplasms in Children as a Factor Inducing Negative Emotions and Toxic Stress in Parents

**DOI:** 10.3390/ijerph191811307

**Published:** 2022-09-08

**Authors:** Grażyna Cepuch, Agnieszka Kruszecka-Krówka, Marzena Samardakiewicz, Agnieszka Gniadek, Agnieszka Micek

**Affiliations:** 1 Nursing and Midwifery Institute, Faculty of Health Sciences, Jagiellonian University Medical College, Kopernika Str. 25, 31-501 Krakow, Poland; 2 Department of Psychology, Faculty of Medical Sciences College Academicum, Medical University of Lublin, Chodźki Str. 7, 20-093 Lublin, Poland

**Keywords:** children with cancer, parents, negative emotions, toxic stress

## Abstract

Parents whose children suffer from cancer experience chronic negative emotions, which may have a detrimental influence on their mental health. The aim of this study, conducted with a group of parents whose children were hospitalized for leukemia or lymphoma, was to assess stress, anxiety, depression, aggression and stress coping strategies as well as the correlations that take place between them. The study was conducted with a group of 101 parents of early school children (aged between 7 and 12) who were hospitalized for cancer in three medical centers in southern Poland. The HADS –M, PSS-10 and COPE questionnaires were used in the study. Mothers were found to experience higher levels of anxiety, depression and stress as compared to fathers. It was more common for men to resort to the strategy of substance use. Socio-demographic variables did not determine the examined emotions, the level of stress and the choice of stress coping strategies, with the exception of the strategy of suppression of competing activities. A positive relationship was found between the incidence of negative emotions and the selected strategies of coping with stress. Early diagnosis of disorders and assessment of parents’ strategies of coping might help to counteract long-term consequences of trauma.

## 1. Introduction

Despite its positive survival prognosis [1,2], hematopoietic and lymphoid cell neoplastic diseases in children place a significant burden on all family members [3], leading to a strong emotional crisis [4] as well as to multidimensional changes in the family system. Such a medical diagnosis provokes various reactions in children’s parents. They are confronted with the danger which their children’s lives are exposed to and they experience fear and anxiety when the side effects of the treatment appear. Not only do they experience a mental burden but also, their child’s illness affects the relationship between them [5,6] including their intimate life [7]. Moreover, parents have to struggle with financial problems and their professional career may be destabilized. Regardless of their socioeconomic status, every family with a child suffering from the disease experiences a significant financial burden. However, the families with a lower financial status are more likely to become seriously impoverished [8]. Such financial pressure has a significant impact on the level of anxiety and depression and also has negative consequences for parents’ physical health [9].

In some parents, cancer diagnosis provokes a strong but transient emotional response, which is followed by adaptation to the new situation. However, in some cases the adaptation process may be disturbed as a consequence of prolonged emotional reactions related to toxic stress, sleep disorders [10,11], anxiety and depressive disorders [12]. Responsibilities and restrictions resulting from taking care of a seriously ill child dramatically limit parents’ opportunities to take up other activities in their free time. It should be noted that activities different than the ones related to the child’s disease act as a buffer and reduce negative consequences of an experienced trauma; their lack or shortage has a negative impact on caregivers’ well-being [13].

In the context of cancer and its consequences for parents’ mental well-being, the ability to cope with stress and negative emotions is becoming more and more important. A family’s ability to cope with a difficult situation is a concept that involves combining cognitive and behavioral functions, in which family resources act synergistically in order to contribute to restoring balance. It is how the family copes with a stressful situation that influences its abilities to adapt to a difficult situation [14,15]. Inefficient coping with a difficult situation has a negative impact, especially on mothers, which may result in increasing distress and may have a detrimental influence on interactions with a child [7]. However, a task-based approach to the child’s disease applied by parents of oncological patients which is based on a change of cognitive perspective leads to an improvement in the functioning in the emotional sphere and to beneficial changes in the family system. Therefore, the assessment of parents’ emotional condition and a quick intervention seem to be crucial for improving both parents’ and child’s well-being. Interventions of the medical team, including the nursing team, might contribute to reducing caregivers’ stress and anxiety, which are induced in the course of the child’s disease and the process of hospitalization [16].

The aim of this study, conducted in a group of parents whose children were hospitalized for leukemia or lymphoma, was to assess stress, anxiety, depression, aggression and stress coping strategies as well as the correlations that take place between them.

## 2. Materials and Methods

### 2.1. Study Group

#### 2.1.1. Study Design

A cross-sectional study was designed to evaluate the prevalence of anxiety, depression and aggression in parents of children diagnosed with leukemia or lymphoma and to assess their level of stress and the methods of coping with it. We used the STROBE checklist for cross-sectional studies when writing our report [17]. A written consent for participation was obtained prior to data collection. The study involved parents who provided parental care to their children during their hospital stay. The privacy and confidentiality of participants was strictly protected. All the information provided by each participant was coded by a number that did not directly identify any individual and all identifying information was coded and removed from all non-numerical data to make it impossible for anyone but the researcher to identify any individual. The study was conducted in accordance with the ethical principles of the Helsinki Declaration. The protocol of the study was approved by the Bioethics Committee of the Jagiellonian University (No. 1072.6120.253.2018). 

#### 2.1.2. Setting and Participants

The study was conducted among parents of children suffering from blood cancer and staying in three leading pediatric cancer treatment centers in southern and eastern Poland (Kraków, Kielce, Lublin) from 2019 to 2021.

In the preliminary selection of the study group, medical records of hospital wards were analyzed in order to select only parents of children aged between 7 and 12 and diagnosed with blood cancer—acute lymphoblastic leukemia (ALL), acute myeloid leukemia (AML) or Hodgkin’s lymphoma (HD). The age selection of the study group was determined by the characteristics of children’s natural development which could affect parents (their understanding of the concept of the disease and the risks involved—asking difficult questions about health condition, treatment, diagnosis or death), the frequency of these diseases and their prognosis in a typical course.

The qualification of parents to the study group was determined by the following criteria: being a parent of a child who meets the conditions specified in the selection of the children’s group, having no other children (the ill child is the only child), declaration of being a full family either as a married couple or domestic partnership, declaration of experiencing no other trauma during the child’s illness (e.g., death in the family, one’s own serious disease, serious disease of a spouse or a loved one, intended separation or divorce, losing a job or any other situation perceived by the respondent as a traumatic one).

Legal guardians who were not children’s parents were excluded from the recruitment. Additionally, a study sample was restricted only to parents of children not diagnosed with infiltration in the nervous system and relapses of the disease, with a completed first full course of chemotherapy or in its final stage, stable health condition (no radical exacerbation due to the disease itself or due to the administered treatment), without chronic comorbidities.

#### 2.1.3. Patient and Public Involvement

The subjects of the study were the children’s parents who were the main source of information. The patients themselves were not involved in the study in any way.

### 2.2. Description of Research Tools

To assess parental anxiety, depression, aggression, the level of stress and how to deal with it, the method of diagnostic survey was applied including the following survey questionnaires:

₋ A self-designed questionnaire that included sociodemographic data (respondent’s age, sex, education, place of residence as well as the child’s age, type of disease, duration of the disease and treatment). The duration of the disease was divided into two periods: 6 months or less and from 6 months up to 1 year. Such a division into 2 periods reflected the types of chemotherapy applied in these diseases: the average, short period of chemotherapy (up to 6 months) and the full, maximum period (over 6 months).
₋ Hospital Anxiety and Depression Scale (HADS), original version developed by Zigmond & Snaith [18]; Polish version adapted by Majkowicz, de Walden-Gałuszko, & Chojnacka-Szawłowska [19]. The tool is commonly used to screen the emotional state of not only ill (somatically, psychosomatically) people during hospitalization, outpatient treatment and at home, but also of their caregivers or partners (in the case of adults). Moreover, the tool is used to assess the emotional state of healthy people, and those at risk of developing anxiety disorder and depression. The questionnaire includes two independent categories used for assessing anxiety and depression in non-psychiatric patients. Each category consists of seven statements. Additionally, the questionnaire assesses irritability/aggression based on two statements. All the answers are given on a 4-point Likert scale (0–3). The final score for anxiety and depression ranges between 0 and 21 points and for aggression, between 0 and 6 points. Scores of anxiety and depression are categorized according to the following cut-off points: The score between 0 and 7 indicate a normal state. The range 8–10 indicates a borderline case, whereas the range 11–21 is considered abnormal.
₋ The Perceived Stress Scale—10—(PSS-10), developed by Cohen, Kamarck & Mermelstein [20]; Polish version adapted by Juczyński & Ogińska-Bulik [21]. The scale consists of 10 questions referring to respondents’ subjective feelings connected with problems and personal experience, behaviors and coping strategies which are assessed on a 5-point scale ranging from 0 (never) to 4 (very often). Before calculating the general indicator of the intensity of perceived stress, changes are introduced in the scores for positively formulated questions (4, 5, 7, 8) following the rule: 0 = 4; 1 = 3; 3 = 1; 4 = 0. The total score is the sum of all scores and ranges between 0 and 40; the higher the score, the higher intensity of perceived stress. The general index is transformed into standardized units and interpreted according to the proprieties characterizing a sten scale. The score ranging from 1 to 4 stens is considered to be low, from 5 to 6 stens—average, and from 7 to 10 stens—high. The score of 10 on the PSS scale is an indicator of assessing one’s own life situation as stressful, unpredictable, beyond control and excessively burdensome.
₋ Coping Orientation to Problems Experienced (COPE), created by Carver, Scheier & Weintraub [22], Polish version adapted by: Juczyński & Ogińska-Bulik [21]—is used to assess typical ways of responding to stressful situations. The COPE questionnaire consists of 60 statements related to 15 coping strategies (4 statements for each). The respondent selects one of the 4 possible responses to each statement: 1—I hardly ever do it, 2—I rarely do it, 3—I often do it, 4—I almost always do it. Each scale is scored separately by adding up the scores obtained for responses referring to the four statements included in the scale. The total score for each scale is then divided by 4, which will produce the result reflecting the frequency with which a given strategy is applied (1—I hardly ever do it, 2—I rarely do it, 3—I often do it, 4—I almost always do it). The following are the names of the scales and the statements assigned to them: 1. *Active coping* (undertaking actions aimed at removing or reducing the stressor or its consequences), 2. *Planning* (considering the ways of coping with the stressor), 3. *Seeking instrumental support* (looking for advice, help or information), 4. *Seeking emotional support* (looking for moral support, sympathy or understanding), 5. *Suppression of competing activities* (avoiding other activities which are not connected with the problem so as to cope with the problem more efficiently), 6. *Turning to religion* (religion as a source of emotional support and guidelines for positive re-evaluating and development), 7. *Positive reinterpretation and development* (finding in a given situation potential for development, perceiving the situation in a more positive light), 8. *Restraint coping* (refraining from acting prematurely, waiting for the right moment), 9. *Acceptance* (accepting the situation as irreversible, as something one has to get used to and learn to live with), 10. *Focus on and venting of emotions* (concern about one’s emotions and the tendency to give vent to them), 11. *Denial* (ignoring, denying that something has happened), 12. *Mental disengagement* (avoiding thinking about the consequences of the situation by engaging in other activities such as sleeping or watching TV), 13. *Behavioral disengagement* (helplessness, resigning from efforts to reach the goals), 14. *Substance abuse* (drinking alcohol or taking other psychoactive substances in order to relieve unpleasant emotions temporarily), 15. *Sense of humor* (joking in order to alleviate unpleasant emotions). The COPE scale is divided into two coping styles by streaming items into the following categories: problem-focused coping (*Religion, Acceptance, Planning, Positive reframing, Active coping, Instrumental support, Emotional support,* and *Humor*) and emotion-focused coping (*Self-distraction, Denial, Venting, Behavioral disengagement, Self-blame* and *Substance abuse*). The scores are based on the sum of the scores of each subscale. These two coping styles were used in the definition of the present operational study for an easy interpretation of a stress management plan for parents [23].

### 2.3. Sample and Setting

An a priori calculated sample size of 101 individuals was planned to detect a medium size difference in the PSS score between 2 independent groups (assuming a size effect equal to 0.6), controlling the probability of Type I and Type II errors at the level of 0.05 and 0.20, respectively. The sample size calculation was performed with the asymptotic relative efficiency method, conservatively allowing for using non-parametric tests.

### 2.4. Data Collection

The research material was collected during direct meetings with parents whose children were staying in the hospital at that time. The nursing staff members were not involved in the study. Participation in the study was voluntary and anonymous. Each parent was informed about the purpose of the study and the possibility to resign from further participation at any time without giving a reason and without bearing any consequences.

### 2.5. Statistical Analysis

The scores of COPE strategies, HADS and PSS were described in terms of quartiles because measures of positions are more appropriate for description of scales than means with standard deviations. Differences in the scores of calculated scales between two groups were compared using the Wilcoxon’s rank sum test. The associations between two categorical variables were examined with the chi-square test of independence after validating the assumption guaranteeing the precision of the approximation. Spearman rank correlations were applied to test the interconnectedness between particular strategies of coping with stress and to test the relationship between the PSS score and categories of HADS: depression, anxiety and aggression. The linear mixed-effects models were fitted to explore the association of depression, anxiety and aggression with strategies of coping with stress and the PSS score. To address the hierarchical structure of the data, a random intercept per each of the three reference hospitals was incorporated allowing for clustering the observations, while the remaining covariates were introduced to the model as fixed effects. General linear mixed regression adjusted to potential confounders was utilized in two variants: (1) including depression, anxiety and aggression in separate models as independent variables as well as (2) incorporating all of them together (after testing for the possible multicollinearity) to verify which category of HADS explains the variance in dependent variable (COPE strategy or PSS score) to the fullest extent. For each model, an analogue of R2 goodness-of-fit metric was calculated. The statistical analyses were performed using the R Software for Windows (R Foundation for Statistical Computing, Vienna, Austria, version 4.0.4). Two-tailed significance level was set at *p* < 0.05.

## 3. Results

### 3.1. Characteristics of the Study Group 

The study group consisted of 101 parents. Women accounted for 60.4% (n = 61) of the respondents. The average children’s age was 9 years (q1 = 7; q3 = 10). The average age of parents was 36 (q1 = 33; q3 = 39) with significantly older females than males (Me = 36; q1 = 34; q3 = 39 vs. Me = 34; q1 = 30; q3 = 39, *p* = 0.028). Male and female parents did not differ regarding the place of living, education, the period of time since the diagnosis of the disease and the type of clinical diagnosis. Table 1 presents the details of the characteristics of the selected study group.

### 3.2. Assessment of the Incidence of Negative Emotions (HADS) and the Level of Perceived Stress (PSS-10) in the Examined Group

In the whole examined group, the median levels (and quartiles) of parental depression, anxiety and aggression were as follows: 10 (q1 = 8; q3 = 14), 12 (q1 = 9; q3 = 14) and 4 (q1 = 3; q3 = 5), consecutively. Females compared to males declared a statistically significantly higher level of depression (*p* = 0.008), anxiety (*p* = 0.004) and aggression (*p* = 0.006). The time since diagnosis did not differentiate the levels of depression, anxiety and aggression (Table 2).

An analysis of the data from the HADS scale for Anxiety and Depression taking into account the division into subcategories (no disorders, borderline disorders and the presence of disorders) showed a statistically significant difference in the disorder prevalence between women and men (*p* = 0.011; *p* = 0.005, respectively). The analysis did not include the Aggression category as it was not divided into subcategories (Table 3).

The level of perceived stress was also higher in woman than in men and the difference was statistically significant both on a point scale (*p* = 0.004) and on a sten scale (*p* = 0.004). The period of time since the child’s diagnosis did not differentiate the level of stress intensity in the examined group of parents; see Table 4.

### 3.3. Assessment of Stress Coping Strategies in Parents (COPE) 

**Figure 1:**
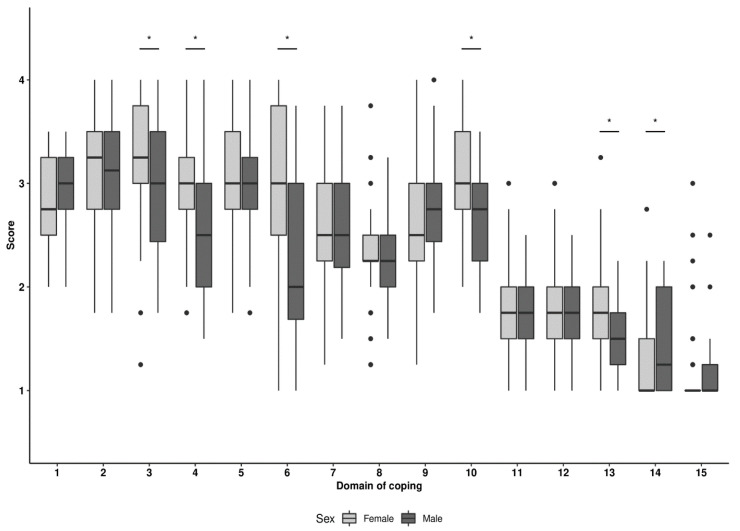
Parents’ stress coping strategies by sex. Note: *—statistical significance.

A statistically significantly higher score was obtained by women as compared to men for the following strategies of coping with stress: *Seeking for instrumental support* (0.008), *Seeking for emotional support* (*p* < 0.001), *Turning to religion* (*p* < 0.001), *Focus on and venting of emotions* (*p* < 0.001) and *Behavioral disengagement* (*p* = 0.002). Only for the strategy *Substance use* was a statistically significantly higher score obtained by men (*p* = 0.015). However, both mothers and fathers presented mainly positive coping strategies (Table S1, Figure 1). In order to better visualize parents’ preferences regarding the strategies of coping with stress, a percentage distribution of the results for particular strategies was presented being coded according to the following scheme: <1.5 points—I hardly ever do it, 1.5–2.5 points—I rarely do it, 2.5–3.5—I often do it, ≥3.5 points—I almost always do it. *Denial, Mental disengagement, Behavioral disengagement, Substance abuse* and *Sense of humor* were strategies chosen by parents more rarely than others; see Figure S1.

Parents of children who were diagnosed 6 or less than 6 months before presented a statistically significantly higher score for the strategy of *Suppression of competing activities*, as compared to parents whose children were diagnosed more than 6 months before (*p* = 0.007). No relations were found between the period of time since the diagnosis of the disease and other strategies of coping with stress—Table S1.

Numerous relationships were found characterized by varying strength and direction between particular strategies of coping with stress in the examined group, which led, among others, to identifying mutual positive correlations between all pairs of strategies such as: *Seeking for instrumental support, Seeking for emotional support, Turning to religion and Focus on and venting of emotion*. On the other hand, the strategies such as *Planning, Seeking for emotional social support* and *Suppression of competing activities* were negatively correlated with *Mental disengagement, Substance use* and *Sense of humor*. Negative correlations were detected also between the strategies: *Substance use* and *Seeking for instrumental social support; Substance use* and *Turning to religion;* as well as *Mental disengagement* and *Turning to religion*. The details of Spearman’s rank correlation analysis between particular stress coping strategies among parents are presented in Table S2. 

### 3.4. Assessment of the Relationship between the Examined Emotional States (HADS) and the Level of Perceived Stress (PSS-10)

A positive correlation of average strength was observed between the level of stress in women and the severity of depression, anxiety and aggression. In the male group, no correlations were found between the level of stress and HADS results in any category. A positive correlation between the level of stress in the examined group of parents and the intensity of examined emotions was observed regardless of the period of time since the diagnosis of the child’s disease—Table 5. 

To assess the relationship between the examined emotional states (HADS) and the level of perceived stress, firstly we built separate models for each of HADS category: depression, anxiety and aggression, adjusted to different sets of covariates. In a fully adjusted model (Model 1) the increase in HADS Depression by 1 point was associated with the increase in the level of stress on PSS by 0.47 points on average (by 0.15 points on PSS stens) with all other variables remaining constant. Compared with those who had no symptoms of depression, parents with depression disorders obtained on average a PSS score 3.69 points higher and by 1.08 stens more on PSS, independently of remaining confounders. In addition, an increase in HADS Anxiety and Aggression of about 1 point was associated with the increase in PSS sequentially about 0.71 (by 0.21 PSS stens) and 1.6 points (by 0.45 PSS stens), on average keeping all other variables constant. Parents with anxiety disorders had on average a PSS score 5.73 points higher (by 1.74 PSS stens), independently of remaining confounders; see Table 6.

After checking which of the HADS categories—depression, anxiety or aggression—had the strongest association with PSS as compared to the others, we incorporated all three subscales into the same model. After including all domains of HADS (as continuous variables) simultaneously in the fully adjusted model, only results for anxiety and children’s age remained significant. An increase in the level of anxiety by 1 point was associated with an increase in the level of stress on the PSS score by about 0.62 on average keeping all other variables constant (β = 0.62, 95% CI: 0.28; 0.95, R2 = 41%). The parents’ stress level decreased with each year of the child’s life by, on average, 0.51 point on the PSS score (β = −0.51, 95% CI: −1.00; −0.02); see Table 7.

### 3.5. Assessment of Correlation between Depression, Anxiety and Aggression (HADS) and Stress Coping Strategies (COPE)

An increase in severity of parents’ depressive disorders was accompanied by higher scores for the following coping strategies applied by parents: *Turning to religion, Restraint coping, Denial* and *Substance use*, no matter whether the model included only the depression category or also other categories, i.e., anxiety and aggression. On the other hand, an increase in the anxiety level was connected with higher sores for strategies such as *Suppression of competing activities, Turning to religion* and *Focus on and venting of emotions*, and with lower scores for *Acceptance* and *Mental disengagement*, regardless of the assumed model. An increase in the level of aggression was followed by a higher score for the strategy of *Behavioral disengagement* (Table 8).

Compared to parents without any syndrome of depression, those with a borderline state of depression and those with depression disorders had, on average, about 0.32 (β = −0.32, 95% CI:−0.60; −0.03) and 0.37 lower (β = −0.37, 95% CI: −0.64; −0.11) score in *Planning strategy*, respectively, independently of all remaining confounders. Parents with depression disorders had also lower score for the strategy *Positive reinterpretation and development* compared to the other respondents (β = −0.29, 95% CI: −0.56; −0.01). However, they had higher score for such strategies as *Turning to religion, Focus on and venting of emotions, Denial, Behavioral disengagement, Substance use* and *Sense of humor*. Parents with anxiety disorders had a higher score for the strategies such as: *Seeking for instrumental support, Turning to religion* and *Focus on and venting of emotions*, but they obtained a lower score for *Acceptance*, compared to the parents with no symptoms of anxiety. (β = −0.39, 95% CI: −0.70; −0.08)—Table 9.

## 4. Discussion

In recent years, there has been a growing interest in the emotional condition of families affected by cancer. Nevertheless, this area requires further research in order to develop an optimal care plan taking into account not only health and development problems of oncological patients but also family and socio-cultural conditions.

The results of studies conducted in various research centers with the application of various research tools indicate a high level of negative emotions as well as toxic stress among parents of children suffering from cancer [12,24,25,26,27,28]. In addition, the results of the current study have shown that a significant percentage of parents struggle with psychoemotional problems, regardless of the duration of the child’s disease. Similar to the findings obtained by Rahmani et al. [4], Al Quadire et al. [12] and Pinquart [29], anxiety was the dominant emotion in the examined group, regardless of parents’ sex, although its frequency was statistically significantly higher in mothers. Anxiety as the background emotion dominating the lives of families struggling with their child’s disease was accompanied by anger, violence, sadness and depression. An analysis of the collected data confirms this relationship. Depressive disorders were diagnosed in more than half of the parents and, which should be noted, they were statistically significantly more frequent in women. In addition, the level of aggression was higher in mothers than in fathers. However, the obtained results should be treated with caution. It cannot be ruled out that men are less likely to show discomfort and psychological dysfunctions than women, and that depression in men tends to be relieved faster than in women [13,30]. Other authors exploring this area confirm the thesis that mothers are more likely to reveal their negative emotions as compared to fathers [24,31].

The incidence of a high level of perceived stress was observed in almost 70% of the examined parents and, additionally, in the group of women it was connected to intensified anxiety, depression and aggression. No comparable relationship was observed in the group of fathers. The differences might have been conditioned not only by individual resources connected with one’s gender, but also by the burden resulting from taking on the role of the main caregiver during hospitalization, which in many cultures [23,32], including the Polish one [25] still remains the domain of women. The factors which contribute to women’s resignation from their professional career include the need to perform the role of mother, a patriarchal social system and the earnings, which are frequently lower than in the case of men. At the same time, mothers who become an important part of a therapeutic team are more vulnerable to emotional and physical overload as compared to fathers [5]. Although Pinquart [33] and Baters [28] indicated a higher level of perceived stress in women as compared to men, Rahmani et al. [4] did not find any significant differences in the incidence of negative emotions depending on parents’ gender. On the other hand, the meta-analysis of van Warmerdam [34] and the study conducted by Mekonnen [5] may lead to a conclusion that there are also other factors which may be responsible for the onset of depression such as single parenthood or lack of support [5]. The current study did not assess these variables. However, it should be noted that even though the status of a full family was an inclusion criterium for taking part in the study, dysfunctions in this area cannot be ruled out. Moreover, an important factor determining the incidence of negative emotions in parents was the fact that they had only one child and, consequently, the focus on his or her disease was much stronger. The differences between the findings obtained in the current study and the reports by other authors may, therefore, be explained not only by methodological differences resulting from the research tools used but also from the inclusion criteria for selecting the study group.

Scientific reports indicate that stress levels in a group of parents of children with cancer varied by the child’s age, as well as the disease severity and duration. Behavioral problems of the child and low parental mental health were the strongest correlates of parenting stress [33]. However, in the current study, the level of stress intensity did not differ between parents of children from various age groups or with a different duration of the disease. Perhaps the selection of the research tool as well as the homogeneity of the examined group might have determined the results of the study. Based on the analyzed scientific literature [34,35,36] and the obtained results, it is possible to demonstrate a relationship between the incidence of negative emotions and stress, including a post-traumatic stress disorder. The observed relationship between emotions and stress including a post-traumatic stress disorder is particularly important as it can also determine the emotional condition of ill children and make them develop a trauma or post-traumatic stress [37]. The psychoemotional condition of parents has a dramatic impact on their ability to fulfill their assigned childcare roles. This, in turn, determines a better therapeutic and developmental effect achieved by children [31,38]. 

The present study also supplied an assessment of stress coping strategies chosen by parents. The vast majority of parents had a tendency to opt for positive strategies, which was also observed in the study presented by Sutan et al. [23]. However, statistically significant differences were observed between women and men in their choices. The most frequently chosen strategies, especially in the female group, were based on *Seeking emotional support, Focusing on and venting of emotions* as well as *Turning to religion*. In the Polish society, searching for support in religion is common and results from the historically conditioned relationship between social life, Catholicism and the institution of the Church as well as from still relatively low cultural diversity of the society. In addition, the studies demonstrated by Sutan [23] and Sharma [32] focus on parents’ choice of the strategy of *Turning to religion*, noting that religious affiliation may determine the choice of stress coping strategies. The choice of Turning to religion as a strategy of coping with stress should be assessed and interpreted by researchers with great caution as it can take two different forms: a positive and a negative one. Positive forms of *Turning to religion* strategy strengthen the relationship with the sacred and create the sense of getting closer to God. However, the negative forms of *Turning to religion* strategy work the opposite way leading to focusing on tension and continuous sense of struggling with guilt and punishment. Dolan et al. [39] emphasize the need of potential screening tests aimed at detecting and responding to negative religious attitudes. *Substance use* was, in turn, the least common strategy among all parents, although it was significantly more frequently chosen by fathers. Parents of children whose duration of the disease was shorter than 6 months were more likely to go for the strategy of *Suppression of competing activities*, as compared to parents whose children suffered from the disease longer, which can be explained by a strong concentration on the child’s health and high motivation to undertake proper treatment. Due to the framework and limitations of the current report, not all strategies of coping with stress which were observed in the examined group of parents were discussed in detail.

Taking into account the findings of the study, it seems essential that medical teams should continuously assess the incidence of anxiety, aggression and the level of stress in parents of children suffering from cancer. Similar suggestions have been put forward by other researchers [31]. The HADS and PSS-10 scales seem to be appropriate screening tools as they may be used by all professionals in a medical team and not only by psychologists. Moreover, these tools are simple and clear for respondents. Regular assessment of parents’ emotional condition and their level of stress is necessary because of their tendency to fluctuate [40]. Interdisciplinary teams should ensure that both patients and their relatives are provided with professional and individual support at every stage of hospitalization [41].

## 5. Strengths, Limitations and Implications of Research

The findings of the study are in line with other research into family functioning and emotions experienced by parents facing their children’s cancer diagnosis. This proves that cancer, even with a good treatment prognosis, inevitably generates considerable stress and negative emotions in parents. The research tools used in the study are widely available, free and easy to interpret. Therefore, they may be used by nurses or doctors as good screening tests. Quick initial detection of potential disorders by members of medical teams who are not psychologists may speed up the identification of parents who require quick professional intervention.

However, the current study has also numerous limitations. Firstly, it was conducted in only three medical centers located in southern Poland. The homogenous group of parents participating in the study is only a small part of all the population of parents whose children struggle with cancer. The study involved a higher number of mothers than fathers, which might have resulted in the lack of statistically significant differences between the genders. Another limitation is the lack of assessment of parents’ emotional condition and their level of stress in the course of their child’s treatment at other time points than the one initially assumed in the study. This makes it difficult to properly examine the trajectories of the intensity of stress, anxiety and depression as well as the coping strategies which are used by respondents.

## 6. Conclusions

Mothers are more vulnerable to anxiety, depression and aggression and they experience a higher intensity of stress in comparison to fathers. The vast majority of respondents tended to adopt positive coping strategies. It was more common for fathers to resort to the strategy of *Substance use* and this difference was statistically significant, whereas women were more likely to choose *Turning to religion, Focusing on emotions* or *Seeking support*. A relationship was found between negative emotions and the type of coping strategies that the respondents chose. Socio-demographic variables and the duration of children’s illness did not determine the examined emotions, the level of stress and the choice of stress coping strategies, with the exception of the strategy of *Suppression of competing activities*. The care quality improvement plan in pediatric oncological departments should focus both on patients and their parents. The key task of the medical team should be an early detection of any possible disorders so as to counteract the long-term consequences of the trauma. The selected research tools can be used for screening tests by all members of a medical team and not only by psychologists.

## Figures and Tables

**Table 1:** Research group profile.

Characteristics of the Study Group	Female (n = 61)	Male (n = 40)	*p* *
	n (%)	n (%)	
**Place of residence**			
City	37 (60.7)	21 (52.5)	0.545
Country	24 (39.3)	19 (47.5)	
**Parents’ education**			
Higher	26 (42.6)	14 (35.0)	0.577
Other than higher	35 (57.4)	26 (65.0)	
**Period of time since the diagnosis**			
≤6 months	31 (50.8)	20 (50.0)	1.000
>6 months	30 (49.2)	20 (50.0)	
**Type of clinical diagnosis**			
Leukemia	38 (62.3)	23 (57.5)	0.784
Lymphoma	23 (37.7)	17 (42.5)	

Note: n—number. * Based on chi-square test of independence.

**Table 2:** The depression, anxiety and aggression scores by parents’ sex and period since the diagnosis of child’s disease.

HADS—Category	Parent’s Sex			*p* *	Period Since the Diagnosis		*p* *
	Total (n = 101)	Female (n = 61)	Male (n = 40)		≤6 Months (n = 51)	>6 Months (n = 50)	
	q2 (q1–q3)	q2 (q1–q3)	q2 (q1–q3)		q2 (q1–q3)	q2 (q1–q3)	
**Depression**	10 (8; 14)	11 (9; 14)	9 (6.75; 11)	**0.008**	10 (8; 14)	10 (7.25; 13.75)	0.555
**Anxiety**	12 (9; 14)	13 (10; 15)	10 (8; 14)	**0.004**	12 (9.5; 14.5)	11 (8.25; 14)	0.192
**Aggression**	4 (3; 5)	4 (3; 5)	4 (3; 4.25)	**0.006**	4 (3; 5)	4 (3; 5)	0.778

Note: n—number; q2, q1, q3—quartiles. * Based on the Wilcoxon rank sum test.

**Table 3:** Parental depression and anxiety according to parents’ sex and period of time since child’s diagnosis by subcategories.

HADS Category		No Symptoms	Borderline State	Disorder	*p* *
		n (%)	n (%)	n (%)	
**Depression**	**Parent’s sex**				
	Female	8 (13.1)	17 (27.9)	36 (59.0)	**0.005**
	Male	15 (37.5)	13 (32.5)	12 (30.0)	
	**Period since the diagnosis**				
	≤6 months	10 (19.6)	16 (31.4)	25 (49.0)	0.742
	>6 months	13 (26.0)	14 (28.0)	23 (46.0)	
**Anxiety**	**Parent’s sex**				
	Female	5 (8.2)	12 (19.7)	44 (72.1)	**0.011**
	Male	8 (20.0)	15 (37.5)	17 (42.5)	
	**Period since the diagnosis**				
	≤6 months	6 (11.8)	12 (23.5)	33 (64.7)	0.667
	>6 months	7 (14.0)	15 (30.0)	28 (56.0)	

Note: n—number. * Based on chi-square test of independence.

**Table 4:** The level of parental stress by sex and period since the diagnosis.

PSS	Parent’s Sex			*p* *	Period Since the Diagnosis		*p* *
	Total (n = 101)	Female (n = 61)	Male (n = 40)		≤6 Months (n = 51)	>6 Months (n = 50)	
	q2 (q1–q3)	q2 (q1–q3)	q2 (q1–q3)		q2 (q1–q3)	q2 (q1–q3)	
**Score**	22 (19; 27)	24 (19; 28)	20.5 (17.75; 25)	**0.004**	23 (19.5; 28)	22 (19; 26)	0.145
**Sten**	7 (6; 9)	8 (6; 9)	7 (6; 8)	**0.004**	8 (6.5; 9)	7 (6; 8)	0.205

Note: n—number; q2, q1, q3—quartiles. * Based on the Wilcoxon rank sum test.

**Table 5:** Correlation between the level of stress and the level of depression, anxiety and aggression by parents’ sex and period since the diagnosis.

PSS/HADS	Depression	Anxiety	Aggression
**PSS Score**			
**Parent’s sex**			
Female (n = 61)	r = 0.46, *p* < 0.001	r = 0.61, *p* < 0.001	r = 0.45, *p* < 0.001
Male (n = 40)	r = 0.07, *p* = 0.685	r = 0.24, *p* = 0.130	r = 0.15, *p* = 0.350
Total (n = 101)	r = 0.39, *p* < 0.001	r = 0.55, *p* < 0.001	r = 0.41, *p* < 0.001
**Period since the diagnosis**			
≤ 6 months (n = 51)	r = 0.42, *p* < 0.001	r = 0.53, *p* < 0.001	r = 0.38, *p* < 0.001
>6 months (n = 50)	r = 0.38, *p* < 0.001	r = 0.58, *p* < 0.001	r = 0.45, *p* < 0.001
**PSS Stens**			
**Parent’s sex**			
Female (n = 61)	r = 0.47, *p* < 0.001	r = 0.6, *p* < 0.001	r = 0.45, *p* < 0.001
Male (n = 40)	r = 0.06, *p* = 0.731	r = 0.28, *p* = 0.079	r = 0.12, *p* = 0.446
Total (n = 101)	r = 0.39, *p* < 0.001	r = 0.54, *p* < 0.001	r = 0.41, *p* < 0.001
**Period since the diagnosis**			
≤6 months (n = 51)	r = 0.39, *p* < 0.001	r = 0.51, *p* < 0.001	r = 0.37, *p* < 0.001
>6 months (n = 50)	r = 0.40, *p* < 0.001	r = 0.58, *p* < 0.001	r = 0.44, *p* < 0.001

**Table 6:** Relationship between depression, anxiety and aggression (as continuous variables and in subcategories) and the level of stress in parents—separate models of multivariable regression for each HADS category.

HADS	PSS Score, B (95% CI)			PSS Stens, B (95% CI)		
	Model 1	Model 2	Model 3	Model 1	Model 2	Model 3
**Depression**						
Per 1 point	0.47 (0.22; 0.73) ***	0.49 (0.25; 0.73) ***	0.5 (0.25; 0.75) ***	0.15 (0.07; 0.22) ***	0.15 (0.07; 0.22) ***	0.15 (0.08; 0.23) ***
No symptoms (n = 23)	0 (ref.)	0 (ref.)	0 (ref.)	0 (ref.)	0 (ref.)	0 (ref.)
Borderline state (n = 30)	1.35 (−1.34; 4.04)	1.53 (−1.03; 4.1)	1.6 (−0.99; 4.18)	0.47 (−0.36; 1.29)	0.51 (−0.27; 1.3)	0.54 (−0.26; 1.34)
Disorder (n = 48)	3.69 (1.17; 6.22) **	3.91 (1.46; 6.35) **	3.97 (1.48; 6.46) **	1.08 (0.3; 1.86) **	1.11 (0.37; 1.86) **	1.16 (0.4; 1.93) **
**Anxiety**						
Per 1 point	0.71 (0.47; 0.94) ***	0.7 (0.47; 0.93) ***	0.71 (0.48; 0.95) ***	0.21 (0.14; 0.29) ***	0.21 (0.14; 0.28)***	0.22 (0.14; 0.29) ***
No symptoms (n = 13)	0 (ref.)	0 (ref.)	0 (ref.)	0 (ref.)	0 (ref.)	0 (ref.)
Borderline state (n = 27)	2.72 (−0.29; 5.73)	2.76 (−0.25; 5.76)	2.57 (−0.41; 5.54)	0.85 (−0.07; 1.77)	0.87 (−0.04; 1.78)	0.83 (−0.08; 1.74)
Disorder (n = 61)	5.73 (2.93; 8.53) ***	5.68 (2.91; 8.45) ***	5.76 (3; 8.53) ***	1.76 (0.9; 2.61) ***	1.74 (0.9; 2.58) ***	1.79 (0.95; 2.63) ***
**Aggression**						
Per 1 point	1.6 (0.71; 2.49) ***	1.44 (0.56; 2.32) **	1.58 (0.74; 2.42) ***	0.45 (0.17; 0.72) **	0.41 (0.14; 0.68) **	0.46 (0.2; 0.72) ***

Note: n—number; ** *p* < 0.001; *** *p* < 0.0001; ref—reference category. Results are presented as unstandardized beta coefficients with 95% confidence intervals: β (95% CI). **Model 1:** parent’s sex [male vs. female (ref)], parent’s age [continuous (per 1 year)], place of residence [city vs. country (ref)], parent’s education [higher vs. other than higher (ref)], period since the diagnosis [ ≤ 6 months vs. >6 months (ref)], child’s disease [lymphoma vs. leukemia (ref)], child’s age [continuous (per 1 year)]; **Model 2**: parent’s sex [male vs. female (ref)], child’s age [continuous (per 1 year)], parent’s education [higher vs. other than higher (ref)], period since the diagnosis [≤6 months vs. >6 months (ref)]; **Model 3:** parent’s sex [male vs. female (ref)], child’s age [continuous (per 1 year)].

**Table 7:** The association between the level of Depression, Anxiety and Aggression (HADS) as continuous variables and the level of stress (PSS score and stens)—model of multivariable regression common for all HADS categories.

Explanatory Variable	PSS Score β (95% CI)	*p*	PSS Stens β (95% CI)	*p*
Depression, per 1 point	0 (−0.31; 0.32)	0.981	0.01 (−0.09; 0.1)	0.880
Anxiety, per 1 point	**0.62 (0.28; 0.95)**	**0.001**	**0.19 (0.08; 0.29)**	**0.001**
Aggression, per 1 point	0.74 (−0.14; 1.63)	0.103	0.18 (−0.09; 0.45)	0.193
Sex, female	0 (ref.)		0 (ref.)	
Sex, male	−1.23 (−3.1; 0.64)	0.201	−0.34 (−0.92; 0.23)	0.246
Parent age	0.17 (−0.04; 0.37)	0.112	0.04 (−0.02; 0.11)	0.165
Place of residence, village or small city	0 (ref.)		0 (ref.)	
Place of residence, large city	0.77 (−0.92; 2.47)	0.372	0.24 (−0.28; 0.76)	0.364
Education, grammar or vocational	0 (ref.)		0 (ref.)	
Education, secondary	0.07 (−2.11; 2.26)	0.947	−0.01 (−0.68; 0.67)	0.985
Education, higher	−0.68 (−2.84; 1.48)	0.538	−0.13 (−0.79; 0.53)	0.703
Time since the diagnosis >6 months	0 (ref.)		0 (ref.)	
Time since the diagnosis ≤6 months	0.73 (−0.94; 2.4)	0.394	0.17 (−0.34; 0.69)	0.515
Disease, leukemia	0 (ref.)		0 (ref.)	
Disease, lymphoma	0.02 (−1.73; 1.76)	0.983	−0.18 (−0.72; 0.35)	0.506
Child’s age	**−0.51 (−1; −0.02)**	**0.043**	**−0.18 (−0.33; −0.03)**	**0.021**

Results are presented as unstandardized beta coefficients with 95% confidence intervals: β (95% CI).

**Table 8:** The association between the level of parental depression, anxiety and aggression and the use of particular coping strategies including selected demographic and clinical characteristics—multivariable regression.

COPE Strategy/Increase per 5 Points	Depression ^a^	Depression ^b^	Anxiety ^a^	Anxiety ^b^	Aggression ^a^	Aggression ^b^
	β (95% CI)	β (95% CI)	β (95% CI)	β (95% CI)	β (95% CI)	β (95% CI)
**1.** **Active coping**	−0.07 (−0.18; 0.04)	−0.03 (−0.18; 0.12)	−0.08 (−0.19; 0.03)	−0.04 (−0.2; 0.11)	−0.21 (−0.58; 0.17)	−0.11 (−0.52; 0.29)
**2.** **Planning**	−0.13 (−0.27; 0.01)	−0.15 (−0.34; 0.04)	−0.07 (−0.21; 0.07)	0.04 (−0.17; 0.24)	−0.12 (−0.61; 0.38)	−0.01 (−0.55; 0.52)
**3.** **Seeking for instrumental support**	0.18 (0.02; 0.34) *	**0.11 (−0.11; 0.33)**	0.18 (0.01; 0.34) *	**0.12 (−0.11; 0.36)**	0.09 (−0.49; 0.66)	−0.19 (−0.8; 0.43)
**4.** **Seeking for emotional support**	0.16 (0.02; 0.30) *	**0.13 (−0.06; 0.32)**	0.13 (−0.01; 0.26)	0.06 (−0.13; 0.26)	−0.03 (−0.51; 0.46)	−0.22 (−0.74; 0.29)
**5.** **Suppression of competing activities**	0.05 (−0.08; 0.18)	−0.15 (−0.31; 0.01)	0.19 (0.07; 0.32) **	0.31 (0.14; 0.49) ***	0.17 (−0.28; 0.61)	−0.12 (−0.58; 0.34)
**6.** **Turning to religion**	0.44 (0.23; 0.65) ***	0.29 (0.02; 0.56) *	0.43 (0.22; 0.64) ***	0.33 (0.04; 0.62) *	**−0.13 (−0.91; 0.65**)	−0.85 (−1.62; −0.09) *
**7.** **Positive reinterpretation and development**	−0.11 (−0.25; 0.03)	−0.06 (−0.25; 0.14)	−0.12 (−0.27; 0.03)	−0.05 (−0.26; 0.16)	−0.37 (−0.87; 0.13)	−0.25 (−0.8; 0.3)
**8.** **Restraint coping**	0.11 (0.00; 0.23) *	0.19 (0.04; 0.34) *	0.01 (−0.1; 0.13)	−0.07 (−0.23; 0.09)	−0.34 (−0.72; 0.05)	−0.4 (−0.81; 0.01)
**9.** **Acceptance**	−0.14 (−0.28; 0) *	**−0.02 (−0.21; 0.17)**	−0.2 (−0.34; −0.06) **	−0.22 (−0.42; −0.02) *	−0.1 (−0.6; 0.4)	0.22 (−0.31; 0.74)
**10.** **Focus on and venting of emotions**	0.26 (0.13; 0.39) ***	**0.07 (−0.11; 0.24)**	0.33 (0.2; 0.46) ***	0.27 (0.09; 0.46) **	0.56 (0.07; 1.05)*	**0.11 (−0.37; 0.6)**
**11.** **Denial**	0.2 (0.09; 0.31) ***	0.16 (0.01; 0.3) *	0.17 (0.06; 0.28) **	**0.09 (−0.07; 0.24)**	0.07 (−0.32; 0.47)	−0.18 (−0.59; 0.24)
**12.** **Mental disengagement**	−0.06 (−0.17; 0.06)	0.11 (−0.03; 0.26)	−0.17 (−0.28; −0.06) **	−0.28 (−0.43; −0.13) ***	0.01 (−0.37; 0.4)	0.3 (−0.1; 0.69)
**13.** **Behavioral disengagement**	0.19 (0.08; 0.3) **	**0.14 (−0.01; 0.29)**	0.16 (0.05; 0.28) **	**0.02 (−0.14; 0.17)**	0.58 (0.2; 0.97) **	0.44 (0.03; 0.85) *
**14.** **Substance use**	0.13 (0.01; 0.24) *	0.19 (0.03; 0.35) *	0.03 (−0.09; 0.15)	−0.14 (−0.3; 0.03)	0.3 (−0.11; 0.71)	0.3 (−0.14; 0.74)
**15.** **Sense of humor**	**0.09 (−0.01; 0.19)**	0.16 (0.02; 0.3) *	0 (−0.1; 0.1)	−0.12 (−0.27; 0.02)	0.09 (−0.26; 0.43)	0.11 (−0.27; 0.49)

Note: * *p* < 0.05; ** *p* < 0.001; *** *p* < 0.0001; ref- reference category. Results are presented as unstandardized beta coefficients with 95% confidence intervals: β (95% CI). ^a^ Adjusted to: parent’s sex [male vs. female (ref)], parent’s age [continuous (per 1 year)], place of residence [city vs. country (ref)], parent’s education [higher vs. other than higher (ref)], period since the diagnosis [≤6 months vs. >6 months (ref)], child’s disease [lymphoma vs. leukemia (ref)], child’s age [continuous (per 1 year)]; ^b^ Adjusted to: parent’s sex [male vs. female (ref)], parent’s age [continuous (per 1 year)], place of residence [city vs. country (ref)], parent’s education [higher vs. other than higher (ref)], period since the diagnosis [≤6 months vs. >6 months (ref)], child’s disease [lymphoma vs. leukemia (ref)], child’s age [continuous (per 1 year)] and two remaining domains of HADS.

**Table 9:** The association between the particular category of depression and anxiety and the use of particular coping strategies—multivariable regression.

COPE Strategy	Depression, β (95% CI)			Anxiety, β (95% CI)		
	No	Borderline State	Disorders	No	Borderline State	Disorders
**1.** **Active coping**	0 (ref)	−0.09 (−0.31; 0.13)	−0.18 (−0.38; 0.03)	0 (ref)	0.14 (−0.12; 0.39)	−0.07 (−0.31; 0.16)
**2.** **Planning**	0 (ref)	−0.32 (−0.6; −0.03) *	−0.37 (−0.64; −0.11) **	0 (ref)	−0.1 (−0.44; 0.24)	−0.21 (−0.52; 0.11)
**3.** **Seeking for instrumental support**	0 (ref)	0.25 (−0.09; 0.59)	0.25 (−0.07; 0.57)	0 (ref)	0.61 (0.24; 0.98) **	0.71 (0.36; 1.05) ***
**4.** **Seeking for emotional support**	0 (ref)	−0.05 (−0.34; 0.23)	0.15 (−0.13; 0.42)	0 (ref)	−0.06 (−0.4; 0.27)	0.12 (−0.19; 0.43)
**5.** **Suppression of competing activities**	0 (ref)	−0.23 (−0.49; 0.04)	−0.1 (−0.35; 0.15)	0 (ref)	0.04 (−0.26; 0.34)	0.23 (−0.05; 0.51)
**6.** **Turning to religion**	0 (ref)	0.18 (−0.26; 0.62)	0.64 (0.22; 1.05) **	0 (ref)	0.38 (−0.13; 0.89)	0.83 (0.35; 1.3) ***
**7.** **Positive reinterpretation and development**	0 (ref)	−0.25 (−0.54; 0.05)	−0.29 (−0.56; −0.01) *	0 (ref)	−0.1 (−0.45; 0.25)	−0.18 (−0.5; 0.15)
**8.** **Restraint coping**	0 (ref)	−0.11 (−0.35; 0.12)	0.07 (−0.15; 0.29)	0 (ref)	0.07 (−0.21; 0.34)	0.05 (−0.21; 0.3)
**9.** **Acceptance**	0 (ref)	−0.1 (−0.39; 0.2)	−0.16 (−0.43; 0.12)	0 (ref)	−0.09 (−0.42; 0.24)	−0.39 (−0.7; −0.08) *
**10.** **Focus on and venting of emotions**	0 (ref)	−0.02 (−0.3; 0.27)	0.28 (0.01; 0.55) *	0 (ref)	−0.03 (−0.35; 0.3)	0.39 (0.09; 0.69) *
**11.** **Denial**	0 (ref)	0.02 (−0.2; 0.24)	0.31 (0.1; 0.52) **	0 (ref)	0.07 (−0.2; 0.35)	0.23 (−0.02; 0.49)
**12.** **Mental disengagement**	0 (ref)	−0.07 (−0.3; 0.16)	0.01 (−0.21; 0.23)	0 (ref)	−0.05 (−0.31; 0.22)	−0.2 (−0.45; 0.05)
**13.** **Behavioral disengagement**	0 (ref)	0.03 (−0.19; 0.24)	0.39 (0.19; 0.6) ***	0 (ref)	−0.03 (−0.3; 0.24)	0.23 (−0.02; 0.48)
**14.** **Substance use**	0 (ref)	0.14 (−0.09; 0.38)	0.32 (0.1; 0.54) **	0 (ref)	0.2 (−0.09; 0.48)	0.21 (−0.06; 0.47)
**15.** **Sense of humor**	0 (ref)	0.06 (−0.14; 0.27)	0.22 (0.03; 0.42) *	0 (ref)	0.15 (−0.1; 0.39)	0.09 (−0.13; 0.32)

Note:* *p* < 0.05; ** *p* < 0.001; *** *p* < 0.0001; ref—reference category. Results are presented as unstandardized beta coefficients with 95% confidence intervals: β (95% CI). All models adjusted to: parent’s sex [male vs. female (ref)], parent’s age [continuous (per 1 year)], place of residence [city vs. country (ref)], parent’s education [higher vs. other than higher (ref)], period since diagnosis [≤6 months vs. >6 months (ref)], child’s disease [lymphoma vs. leukemia (ref)], child’s age [continuous (per 1 year)].

## Data Availability

Not applicable.

## Supplementary Materials

The following supporting information can be downloaded at https://www.mdpi.com/article/10.3390/ijerph191811307/s1, Table S1: Parents’ stress coping strategies by sex and period since diagnosis, Table S2: Correlations between particular strategies of coping with stress chosen by parents, Figure S1: Parents’ stress coping strategies by sex.
