# Supplementary material for: Hematopoietic and Lymphoid Cell Neoplasms in Children as a Factor Inducing Negative Emotions and Toxic Stress in Parents
Source: Int J Environ Res Public Health. 2022 Sep 8;19(18):11307. doi: 10.3390/ijerph191811307 (PMC9516952; doi:10.3390/ijerph191811307)
Supplement: Supplementary file 1 [file ijerph-19-11307-s001.zip › ijerph-1851892-supplementary tables.pdf]

Table S1. Parents' stress coping strategies by sex and period since the diagnosis.

COPE strategy	Parent's sex		p*	Period since the diagnosis		p*
	Female (N=61)	Male (N=40)		≤6 months (N=51)	>6 months (N=50)	
	q2(q1-q3)	q2(q1-q3)		q2(q1-q3)	q2(q1-q3)	
**1. Active coping**	2.75 (2.5; 3.25)	3 (2.75; 3.25)	0.179	3 (2.75; 3.25)	2.75 (2.5; 3.19)	0.085
**2. Planning**	3.25 (2.75; 3.5)	3.12 (2.75; 3.5)	0.966	3.25 (3; 3.5)	3 (2.75; 3.5)	0.187
**3. Seeking for instrumental support**	3.25 (3; 3.75)	3 (2.44; 3.5)	**0.008**	3.25 (3; 3.5)	3 (2.75; 3.5)	0.335
**4. Seeking for emotional support**	3 (2.75; 3.25)	2.5 (2; 3)	**<0.001**	2.75 (2.5; 3.25)	2.88 (2.5; 3.25)	0.842
**5. Suppression of competing activities**	3 (2.75; 3.5)	3 (2.75; 3.25)	0.863	3.25 (2.88; 3.5)	3 (2.75; 3.25)	**0.007**
**6. Turning to religion**	3 (2.5; 3.75)	2 (1.69; 3)	**<0.001**	3 (1.88; 3.75)	3 (2; 3.19)	0.498
**7. Positive reinterpretation and development**	2.5 (2.25; 3)	2.5 (2.19; 3)	0.771	2.5 (2.25; 3)	2.75 (2.25; 3)	0.628
**8. Restraint coping**	2.25 (2.25; 2.5)	2.25 (2; 2.5)	0.251	2.25 (2.12; 2.5)	2.25 (2; 2.5)	0.600
**9. Acceptance**	2.5 (2.25; 3)	2.75 (2.44; 3)	0.483	2.75 (2.25; 3)	2.75 (2.25; 3)	0.557
**10. Focus on and venting of emotions**	3 (2.75; 3.5)	2.75 (2.25; 3)	**<0.001**	3 (2.75; 3.5)	2.88 (2.5; 3.25)	0.274
**11. Denial**	1.75 (1.5; 2)	1.75 (1.5; 2)	0.338	1.75 (1.5; 2)	1.75 (1.5; 2)	0.983
**12. Mental disengagement**	1.75 (1.5; 2)	1.75 (1.5; 2)	0.744	1.75 (1.5; 2)	1.75 (1.5; 2)	0.674
**13. Behavioral disengagement**	1.75 (1.5; 2)	1.5 (1.25; 1.75)	**0.002**	1.75 (1.25; 2)	1.5 (1.25; 1.75)	0.166
**14. Substance use**	1 (1; 1.5)	1.25 (1; 2)	**0.015**	1 (1; 1.5)	1 (1; 1.75)	0.234
**15. Sense of humor**	1 (1; 1)	1 (1; 1.25)	0.205	1 (1; 1)	1 (1; 1.25)	0.507

q2, q1, q3 – quartiles.

*based on the Wilcoxon rank sum test.

Table S2. Correlations between particular strategies of coping with stress chosen by parents.

COPE strategy	1	2	3	4	5	6	7	8	9	10	11	12	13	14	15
**1**	1.00***	0.48***	0.23*	0.07	0.14	0.00	0.08	0.08	0.15	-0.05	-0.12	-0.08	-0.21*	-0.29**	-0.10
**2**	0.48***	1.00***	0.19	0.06	0.46***	-0.05	0.28**	-0.06	0.13	0.10	0.00	-0.24*	-0.25*	-0.34***	-0.29**
**3**	0.23*	0.19	1.00***	0.42***	0.15	0.39***	0.14	0.09	0.09	0.42***	0.03	-0.21*	-0.09	-0.20*	-0.24*
**4**	0.07	0.06	0.42***	1.00***	-0.01	0.50***	0.15	0.16	-0.10	0.51***	0.30**	-0.17	0.34***	-0.29**	-0.04
**5**	0.14	0.46***	0.15	-0.01	1.00***	0.05	-0.01	0.12	-0.23*	0.29**	0.02	-0.34***	-0.07	-0.21*	-0.30**
**6**	0.00	-0.05	0.39***	0.50***	0.05	1.00***	0.10	0.32***	-0.18	0.41***	0.08	-0.25*	0.24*	-0.29**	-0.11
**7**	0.08	0.28**	0.14	0.15	-0.01	0.10	1.00***	0.04	0.16	-0.01	-0.01	0.22*	-0.11	-0.02	0.08
**8**	0.08	-0.06	0.09	0.16	0.12	0.32***	0.04	1.00***	-0.11	0.34***	0.09	0.07	0.13	-0.14	0.08
**9**	0.15	0.13	0.09	-0.10	-0.23*	-0.18	0.16	-0.11	1.00***	-0.32**	-0.27**	0.26**	-0.24*	0.10	0.04
**10**	-0.05	0.10	0.42***	0.51***	0.29**	0.41***	-0.01	0.34***	-0.32**	1.00***	0.31**	-0.29**	0.25*	-0.35***	-0.15
**11**	-0.12	0.00	0.03	0.30**	0.02	0.08	-0.01	0.09	-0.27**	0.31**	1.00***	-0.02	0.46***	0.25*	0.42***
**12**	-0.08	-0.24*	-0.21*	-0.17	-0.34***	-0.25*	0.22*	0.07	0.26**	-0.29**	-0.02***	1.00***	0.10	0.39***	0.27**
**13**	-0.21*	-0.25*	-0.09	0.34***	-0.07	0.24*	-0.11	0.13	-0.24*	0.25*	0.46***	0.10	1.00***	0.08	0.29**
**14**	-0.29**	-0.34***	-0.20*	-0.29**	-0.21*	-0.29**	-0.02	-0.14	0.10	-0.35***	0.25*	0.39***	0.08	1.00***	0.33***
**15**	-0.10	-0.29**	-0.24*	-0.04	-0.30**	-0.11	0.08	0.08	0.04	-0.15	0.42***	0.27**	0.29**	0.33***	1.00***

***p<0.001; **p<0.01; *p<0.05; 1. Active coping, 2. Planning, 3. Seeking for emotional social support, 4.Seeking for instrumental social support, 5. Suppression of competing activities, 6.Turning to religion, 7. Positive reinterpretation and development, 8. Restraint coping, 9. Acceptance, 10. Focus on and venting of emotions, 11. Denial, 12. Mental disengagement, 13. Behavioral disengagement, 14. Substance use, 15. Sense of humor.
