# Supplementary figures and images for: Hematopoietic and Lymphoid Cell Neoplasms in Children as a Factor Inducing Negative Emotions and Toxic Stress in Parents
Source: Int J Environ Res Public Health. 2022 Sep 8;19(18):11307. doi: 10.3390/ijerph191811307 (PMC9516952; doi:10.3390/ijerph191811307)

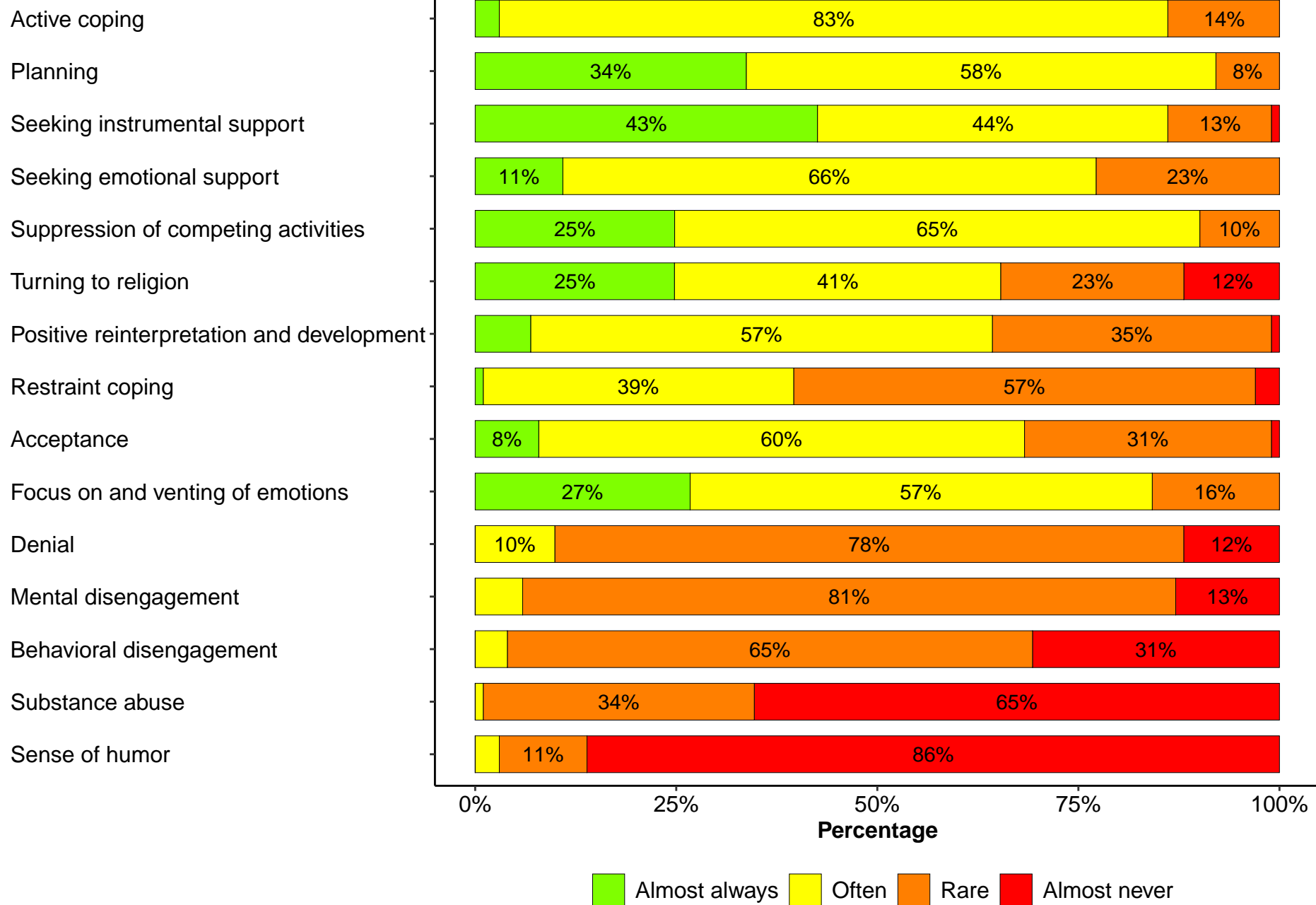

Supplement: Supplementary file 1 [file ijerph-19-11307-s001.zip › Figure S1-Parentsí» stress coping strategies by sex.pdf]
